# Safety of influenza vaccine during COVID-19

**DOI:** 10.1017/cts.2020.543

**Published:** 2020-09-17

**Authors:** Joe G. Zein, Georgina Whelan, Serpil C. Erzurum

**Affiliations:** 1 Respiratory Institute, Cleveland Clinic, Cleveland, OH 44106, USA; 2 Cleveland Clinic Lerner College of Medicine of Case Western Reserve University, Cleveland Clinic, Cleveland, OH 44106, USA

**Keywords:** Translational science, influenza vaccine, COVID-19, outcome, seasonal influenza

Epidemic curves seen during the 1918 influenza pandemic demonstrated multiple resurgent waves caused by the lifting of control measures as the number of new infections decreased [1]. This suggests that the second wave of coronavirus disease 2019 (COVID-19), caused by severe acute coronavirus-2 (SARS-CoV-2), is possible in the fall of 2020. The convergence with influenza season could result in significant morbidity and mortality among susceptible individuals, such as elderly individuals and those with comorbidities. Little is known about coinfection with SARS-CoV-2 and influenza virus, or the interaction between vaccination against influenza and SARS-CoV-2. Last year’s influenza vaccine was administered in 2019 antecedent to the SARS-CoV-2 pandemic, offering an opportunity to test the association between influenza vaccination and COVID-19 incidence and severity. Patients were considered to have worse outcomes from severe COVID-19 if they required hospitalization, were admitted to the intensive care unit (ICU), or died during hospitalization.

We analyzed patients (*n* = 18,868) tested for COVID-19 at the Cleveland Clinic Health System in Ohio and Florida between March 8 and April 15, 2020 [2]. Among these, we excluded 5648 patients who were vaccinated against influenza prior to 2019, but not vaccinated in 2019, in order to exclude bias related to remote vaccination. Among the remaining cohort, 4138 patients who received unadjuvanted influenza vaccination in the fall of 2019 or winter of 2020 were compared to the 9082 who had never received influenza vaccination [2].

An overlap propensity score weighting method was used to control for observed covariate differences between patients who did and did not receive influenza vaccination in 2019. The propensity score for each individual is the predicted probability of receiving influenza vaccination from a nonparsimonious logistic regression model using the covariates listed as clinical characteristics in Table 1. The overlap propensity score weighting method was then applied to directly compare weighted groups on the outcomes of interest [3]. All statistical analyses were conducted with R, version 4.0.1 (R Project for Statistical Computing, Vienna, Austria). A detailed description of the cohort and the statistical method has been reported previously [2].

**Table 1.** Clinical characteristics and outcome of all individuals in the cohort and in the subgroup of patients tested positive for SARS-CoV-2

	All tested individuals			SARS-CoV-2-positive		
	Never vaccinated	Vaccinated in 2019	*p*	Never vaccinated	Vaccinated in 2019	*p*
Clinical characteristics	(*n* = 9082)	(*n* = 4138)		(*n* = 1125)	(*n* = 309)	
Age – year	49.3 [34.6, 62.9]	61.5 [46.9, 72.0]	<0.001	52.7 [38.6, 64.1]	63.3 [49.2, 73.4]	<0.001
Race – no (%)			<0.001			<0.001
White	5985 (65.9)	3050 (73.7)		686 (61.0)	203 (65.7)	
Black	1695 (18.7)	833 (20.1)		246 (21.9)	91 (29.4)	
Other	1402 (15.4)	255 (6.2)		193 (17.2)	15 (4.9)	
Male sex – no (%)	4050 (44.6)	1651 (39.9)	<0.001	593 (52.7)	152 (49.2)	0.30
Non-Hispanic ethnicity – no (%)	7986 (87.9)	3974 (96.0)	<0.001	893 (79.4)	298 (96.4)	<0.001
BMI – kg/m^2^	28.6 [24.4, 33.6]	29.0 [24.8, 34.9]	0.002	29.7 [26.1, 34.0]	30.0 [25.0, 35.5]	0.66
Smoking – no (%)			<0.001			<0.001
Current smoker	1481 (16.3)	504 (12.2)		64 (5.7)	15 (4.9)	
Former smoker	1625 (17.9)	1684 (40.7)		178 (15.8)	123 (39.8)	
Nonsmoker	5976 (65.8)	1950 (47.1)		883 (78.5)	171 (55.3)	
Median annual income – USD	57,250.0 [42,500.9–74,812.2]	59,390.0 [41,635.0–79,201.0]	0.005	58,429.0 [45,161.0–76,719.0]	60,000.0 [43,097.0–81,953.0]	0.91
Exposure to COVID-19 – no (%)	4805 (52.9)	1923 (46.5)	<0.001	825 (73.3)	203 (65.7)	0.01
Family history of COVID-19 – no (%)	4452 (49.0)	1849 (44.7)	<0.001	795 (70.7)	205 (66.3)	0.16
Coexisting conditions – no (%)						
COPD	517 (5.7)	689 (16.7)	<0.001	39 (3.5)	40 (12.9)	<0.001
Asthma	1433 (15.8)	1195 (28.9)	<0.001	121 (10.8)	66 (21.4)	<0.001
Diabetes	1288 (14.2)	1493 (36.1)	<0.001	177 (15.7)	111 (35.9)	<0.001
Hypertension	2885 (31.8)	2677 (64.7)	<0.001	387 (34.4)	205 (66.3)	<0.001
Coronary artery disease	673 (7.4)	998 (24.1)	<0.001	71 (6.3)	57 (18.4)	<0.001
Congestive heart failure	551 (6.1)	880 (21.3)	<0.001	49 (4.4)	61 (19.7)	<0.001
Cancer	848 (9.3)	1149 (27.8)	<0.001	72 (6.4)	71 (23.0)	<0.001
Connective tissue disease	795 (8.8)	1003 (24.2)	<0.001	69 (6.1)	46 (14.9)	<0.001
Long-term medications – no (%)						
NSAIDs	1659 (18.3)	1459 (35.3)	<0.001	189 (16.8)	108 (35.0)	<0.001
Glucocorticoids	1066 (11.7)	1350 (32.6)	<0.001	67 (6.0)	66 (21.4)	<0.001
ACE inhibitors	512 (5.6)	565 (13.7)	<0.001	74 (6.6)	52 (16.8)	<0.001
ARB	374 (4.1)	439 (10.6)	<0.001	70 (6.2)	41 (13.3)	<0.001
Laboratory measurements						
Platelet count – (x 10^9^/L)	239.0 [188.0, 298.0]	233.0 [176.0, 301.0]	0.005	198.0 [160.0, 250.0]	196.0 [157.0, 251.5]	0.66
Eosinophil count – (cells/μL)	70.0 [30.0, 170.0]	80.0 [30.0, 190.0]	0.001	30.0 [30.0, 30.0]	30.0 [30.0, 30.0]	0.55
Lymphocyte count – (10^9^/μL)	1.4 [0.9, 2.1]	1.2 [0.8, 1.9]	<0.001	1.1 [0.7, 1.5]	0.9 [0.6, 1.3]	0.011
Neutrophil count – (10^9^/μL)	5.6 [3.7, 8.7]	5.9 [3.9, 8.9]	0.086	3.9 [2.9, 5.5]	4.1 [2.8, 6.5]	0.33
Hemoglobin – (g/dL)	13.2 [11.5, 14.6]	12.2 [10.0, 13.9]	<0.001	13.6 [12.1, 14.9]	13.30 [11.7, 14.6]	0.07
Albumin – (g/dL)	4.00 [3.50, 4.35]	3.80 [3.40, 4.20]	<0.001	3.80 [3.52, 4.10]	3.70 [3.40, 4.00]	0.10
Total bilirubin – (mg/dL)	0.4 [0.3, 0.7]	0.5 [0.3, 0.7]	0.277	0.4 [0.3, 0.6]	0.4 [0.3, 0.7]	0.07
ALT – (IU/L)	21.0 [14.0, 34.0]	19.0 [13.0, 30.00]	<0.001	26.0 [17.0, 40.0]	22.0 [15.0, 37.8]	0.23
Creatinine – (mg/dL)	0.90 [0.73, 1.15]	0.99 [0.76, 1.41]	<0.001	0.97 [0.80, 1.22]	1.09 [0.82, 1.42]	0.04
Outcome – no (%)						
Positive SARS-CoV-2 test	1125 (12.4)	309 (7.5)	<0.001			
Hospitalization				192 (17.1)	127 (41.1)	<0.001
ICU admission				77 (6.8)	43 (13.9)	<0.001
Hospital mortality				32 (2.8)	20 (6.5)	0.01

Continuous data are presented as median [IQR]. BMI stands for body mass index; USD for US dollar; COPD for chronic obstructive pulmonary disease; NSAIDS for nonsteroidal anti-inflammatory drugs; ACE for angiotensin-converting enzyme; ARB for angiotensin receptor blocker; and ICU for Intensive Care Unit. “Vaccinated” reflects influenza vaccination.

Covariates listed as clinical characteristics were included in the propensity score.

Laboratory measurement were done on peripheral blood collected at the time of SARS-CoV-2 testing.

Two unadjuvanted split virion, inactivated, quadrivalent influenza vaccines were provided to patients in 2019. A high-dose vaccine was given to patients 65 years and older.

Demographics and clinical characteristics are shown in Table 1. Compared to individuals who never received influenza vaccination, those vaccinated in 2019 were older, had a higher body mass index, and a higher income (Table 1). Vaccinated individuals were more likely to be women and of non-Hispanic ethnicity. They also reported more comorbidities and required more medications. Peripheral blood laboratory measurements done at the time of SARS-CoV-2 testing were also significantly different between the two groups (Table 1).

Unadjusted analysis shows that vaccinated individuals were less likely to test positive for SARS-CoV-2 (Table 1). Among individuals with a positive SARS-CoV-2 test, patients previously vaccinated for influenza in 2019 were more likely to be hospitalized. Once hospitalized, they were more likely to be admitted to the ICU and die during hospitalization. The increased risk for worse hospital outcomes was not related to influenza vaccination in the adjusted analysis. Using overlap propensity score weighting, influenza vaccination was unrelated to the incidence of SARS-CoV-2 infection (adjusted OR [95% CI]: 0.79 [0.62–1.00]). Among individuals with COVID-19 (*n* = 1434), influenza vaccination (*n* = 309) did not impact risk for hospitalization (adjusted OR [95% CI]: 1.29 [0.72–2.31]), ICU admission (adjusted OR [95% CI]: 0.65 [0.22–1.79]), or hospital mortality (adjusted OR [95% CI]: 0.98 [0.39–2.43]).

Overall, our analysis demonstrates that influenza vaccination does not increase the incidence of COVID-19 or worsen the related morbidity or mortality. This is consistent with the prevailing evidence that the influenza vaccine is safe and serious adverse events, such as Guillain-Barré syndrome, are rare [4]. Although our data is reassuring, many uncertainties deserve further consideration. Surveillance data needs to be prospectively collected in fall 2020 to study the outcome of concurrent SARS-CoV-2 and influenza infection, and to assess any interaction between influenza vaccinations, a newly developed vaccine against coronavirus, influenza, and COVID-19 infection. The effect of influenza vaccines, and adjuvanted vaccines in particular, on Th17 immune responses in coronavirus immunopathology and on vaccine-induced immune enhancement [5] is unknown and needs to be closely monitored. Occasionally, non-neutralizing antibodies following influenza vaccination or infection have amplified disease severity following a heterologous influenza challenge. In humans, the 2008–2009 seasonal trivalent inactivated influenza vaccine was associated with increased pandemic H1N1 disease severity [6, 7]. This might be relevant in fall 2020, as new avian-like H1N1 swine influenza virus with 2009 pandemic viral genes have been recently reported [8]. However, based on our data, influenza vaccine is not associated with increased pandemic COVID-19 severity, providing reassurance that the vaccination strategy for global influenza should proceed as planned during the COVID-19 pandemic.
